# Genetic variants in the *SIRT6* transcriptional regulatory region affect gene activity and carcass quality traits in indigenous Chinese beef cattle (*Bos taurus*)

**DOI:** 10.1186/s12864-018-5149-0

**Published:** 2018-11-01

**Authors:** Lin-sheng Gui, Sayed Haidar Abbas Raza, Matthew Garcia, Yong-gang Sun, Irfan Ullah, Yin-cang Han

**Affiliations:** 1 State Key Laboratory of Plateau Ecology and Agriculture, Qinghai University, Xining, Qinghai Province 810016 People’s Republic of China; 2 College of Agriculture and Animal Husbandry, Qinghai University, Xining, Qinghai Province 810016 People’s Republic of China; 3 College of Animal Science and Technology, Northwest A&F University, Yangling, Shaanxi 712100 People’s Republic of China; 4 Utah State University, School of Animal Dairy and Veterinary Sciences, Logan, UT 84322 USA; 5 Academy of Animal Science and Veterinary Medicine, Qinghai University, Xining, Qinghai Province 810016 People’s Republic of China; 6 College of Bio-medical engineering Chongqing University Chongqing, Shapingba 400044, People’s Republic of China

**Keywords:** Transcription factor, SIRT6, Fat deposition, Expression pattern

## Abstract

**Background:**

The aim of this study was to analyze potential influences of polymorphisms within the regulatory region of the bovine SIRT6 gene on carcass quality traits. Expression analyses suggested that SIRT6 gene is predominately expressed in kidney, compared with other tissues. In 535 indigenous Chinese beef cattle, two novel single nucleotide polymorphisms (SNPs) were identified within the promoter region of the SIRT6 gene.

**Results:**

Association analysis indicated that G allele of the c.-1100 A > G had a positive effect on fat deposition, and the Hap4/4 diplotype had more favourable results than other dipoltypes with respect to the evaluation of carcass quality traits. Furthermore, promoter activity associated with the Hap3 haplotype was measured at higher levels than the Hap1 haplotype, which would be in agreement with the previously described association analysis.

**Conclusion:**

The SIRT6 promoter variants significantly affect transcriptional levels and subsequently significantly influence bovine intramscular fat content.

## Background

SIR2 and its homologs, termed sirtuins, are members of the class III nicotinamide adenine dinucleotide-dependent deacetylase family [1]. Seven homologues of SIR2, have been designated as SIRT1–7 with various cellular localization and carboxyl termini in mammals [2]. Among the sirtuins, Sirt6, mainly a protein associated with nuclear chromatin, has distinct roles in metabolism, stress resistance and lifespan [3].

Previous studies demonstrated that SIRT6 gene could deacetylate histone H3K9, and modulate the expression level of associated metabolic genes [4]. More specifically, SIRT6 knockdown cells promoted glycolysis via improved activity of Hif1α [5]. The expressions of genes consisted of lipid and glycolysis metabolism were modified by the knockdown of SIRT6 in liver. This was associated with striking phenotypes, including under-size and delayed bone mineralization [6]. Similarly, mice with neural-specific deletion of SIRT6 exhibited somatotropic attenuation associated with reduced growth hormone (GH) levels [7]. In addtion, SIRT6 gene controls cholesterol homeostasis of mice, and negatively regulates lipogenic transcription factors (i.e., SREBP1 and SREBP2) via promoting their phosphorylation [8]. In response to fasting, transgenic mice overexpressing SIRT6 gene attenuated excess fat deposition due to the reduction of PPARγ gene [9]. These results revealed that SIRT6 gene acted as a critical enzyme for the maintaining of lipid metabolism, which may be closely related to fat deposition in mammals.

Through modifying transcription factor binding sites, sequence variation within promoter and other regulatory regions of gene may impact expression level, and influence phenotypes [10]. Previously, four SNPs were identified within the bovine SIRT6 gene, and correlated with carcass quality traits [11]. However, functional mutations within the promoter region of bovine SIRT6 have not been reported. The current study was aimed at analyzing the relationship between promoter polyorphisms and fat deposition in Chinese indigenous cattle.

## Methods

### Ethics approval

All animal experiments were conducted according to the guidelines established by the regulations this work was performed at a farm in the Department of Animal Sciences and Technology at Qinghai University, China. Ethical approval for this study was obtained from the Ethical Committee of Qinghai University. The procedures were approved by the Ethical Committee of China Animal Care Qinghai University.

### Ontogenic expression

**Fig. 1:**
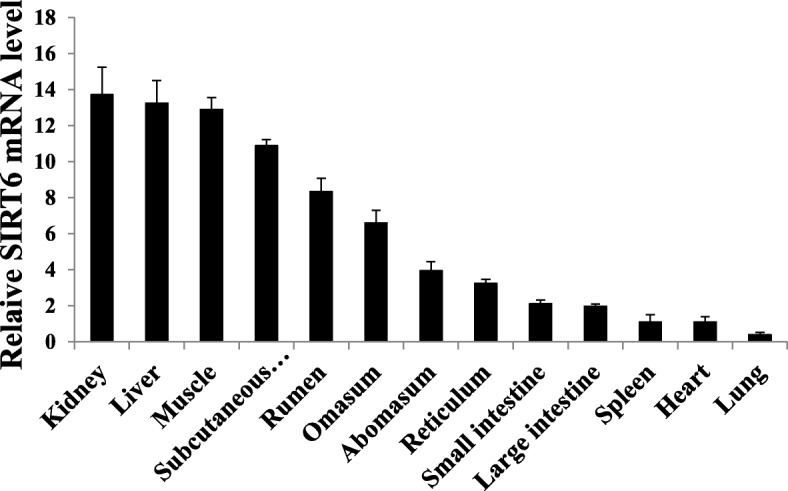
Tissue expression analysis of Qinchuan cattle SIRT6 mRNA

As is shown in Fig. 1, 13 tissues and organs were collected from three purebred bulls of 2 years old of the Qinchuan cattle. Total RNA were performed to reverse transcription using the PrimeSriptTM RT reagent kit (TaKaRa, Dalian, China) based on its recommended procedure.

In this study, two housekeeping genes were used: β-actin (AY141970.1) and GAPDH (NM_001034034). The qPCR was conducted by the Applied Biosystems 7500 Fast Real-Time PCR System (Applied Biosystems, USA) with the SYBR® Premix Ex TaqTM kit (TaKaRa, Dalian, China). The oligonucleotide primers used for the qPCR were designed with Primer Premier 5.0 software (Table 1). The relative results were computed with the 2^-△△Ct^ method [12].

**Table 1:** Primers used in this study

Name	Function	Primer Sequence (5′ to 3′)	Tm (°C)	Product Length	Amplified Region
SIRT6	qPCR	CAACCTGGAGAAATACCGTCTT	61.0	166 bp	400–565
		CAGTCCTTTTTCCTTCAGCAG			
β-actin	Reference	CACCAACTGGGACGACAT	61.0	202 bp	320–521
		ATACAGGGACAGCACAGC			
GAPDH	Reference	CCAACGTGTCTGTTGTGGAT	61.0	80 bp	778–857
		CTGCTTCACCACCTTCTTGA			
Primer A	SNP detection	GAGACGGCCAGGAAGGAC	62.7	320 bp	71 – − 249
		CTGAACGAGGAAACAACG			
Primer B	SNP detection	GACCCCTTCGTCCCCTCAAA	58.5	1367 bp	− 558 – − 1925
		GGGTGGGAAGAGCCAGTAGC			
Primer- c.-84 C > T	SNP genotyping	GAGGTAAGTGGGCGTCAG	60.5	357 bp	− 312 – 45
		CACCAAAGGGAACAATAAAG			
Primer- c.-1100 A > G	SNP genotyping	CCTCAGCTCCCTCCCTCCTAC	62.7	148 bp	− 1218 – − 1070
		CATGATCAGGTGTCAGGGTTGAAT			

### Sample and data collection

Total 535 adult, female individuals between 18 and 24 months old, which were selected from Yangling Shaanxi Province, China. The blood samples were obtained from the jugular vein. Then genomic DNA was isolated from blood samples, stored at − 80 °C until subsequent analyses. Carcass quality traits (backfat thickness, ultrasound loin muscle area and intramuscular fat content) [13], were obtained from each individual.

### Genotyping

As is presented in Table 1, the primers of bovine SIRT6 gene were designed according to the published gene sequence (AC_000164.1). Each PCR reaction was done in a 30 μL reaction mixture containing 50 ng of pooled genomic DNA, 10 pM of primer, 15 μL 2 × Reaction Mix, and 0.3 U Golden DNA polymerase (Tiangen Biotech, Beijing, China). The cycling protocol was performed according to the method of Gui et al. [11].

Two SNPs were detected in the promoter region of the SIRT6, named c.-84 C > T and c.-1100 A > G, respectively. Based on the sequence information, the *ApaLI* and *AgsI* restriction enzymes were utilized to digest PCR products for genotype. The electrophoresis on a 2.5% agarose gel was employed to identify the digested products, which were stained with ethidium bromide.

### Luciferase activity assay

Previous methods were adopted to culture 3 T3-L1 cells [14]. A DNA fragment ranging from − 1224 to + 56 in the SIRT6 gene and encompassing the two polymorphic sites (-84C > T and -1100A > G) was amplified using a forward primer including a *KpnI* site (CGGGGTACC) and reverse primer including a *BglII* site (GGAAGATCT). Use of the dual-luciferase reporter assay standard procedure, the activity of firefly luciferase activity and Renilla luciferase were observed in 3 T3-L1 cells. All experiments were performed in triplicate and repeated twice.

### Statistical analysis

Evaluation of linkage disequilibrium (LD) was conduted by the HAPLOVIEW software (Version 3.32). The general linear model was used for the evaluation of SNP-phenotype association. The equation was as follows: *Y*_*ijk*_ = *u* + *G*_*i*_ + *S*_*j*_ + *A*_*k*_ + *e*_*ijk*_, where *Y*_*ijk*_ were the phenotypic observations; *μ* was the averaged values, *G*_*i*_ was the fixed effect of genotype, *S*_*j*_ was the random effect of sire, *A*_*k*_ was fixed effect of age, and *e*_*ijk*_ was the residual effect.

All values were presented as the mean ± SE. The difference between groups (gene expression levels of qPCR between tissues and relative luciferase activities between different constructs) was analyzed with the two-tailed *t* test.

## Results

### Expression profile

As shown in Fig. 1, bovine SIRT6 gene was ubiquitously expressed in various tissues and organs, with predominant expression level in the kidney, liver, muscle and subcutaneous fat. Whereas, SIRT6 gene had relatively low level in abomasum, reticulum, spleen, heart, lung, small and large intestine.

### Single marker association analysis

**Fig. 2:**
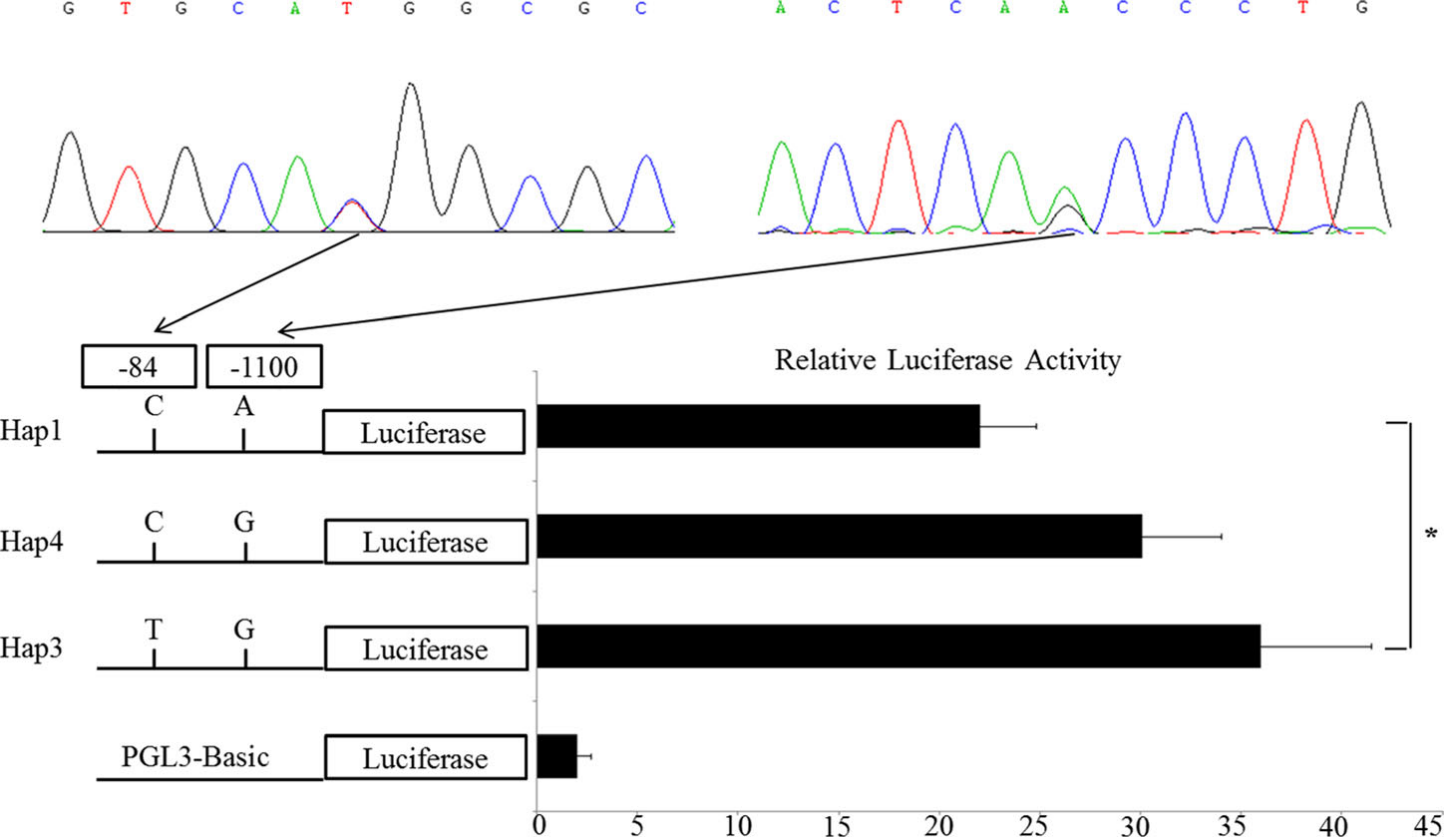
Transcriptional activity of the three haplotype constructs. The obtained data was determined relative to the activity of the empty pGL3 basic plasmid. The mean ± Std indicated the the mean values and standard deviations

Sequencing of PCR products from genomic DNAs revealed two polymorphisms within the SIRT6 promoter, named c.-84 C > T and c.-1100 A > G, respectively (Fig. 2). Based on the sequence information, the *ApaLI* and *AgsI* restriction enzymes were utilized to digest PCR products for genotype.

As shown in Table 2, the backfat thickness and intramuscular fat content of individuals that inherited genotype -1100GG were significantly higher than those of individuals with the AA or GA genotype -1100AA (*P* < 0.05). Whereas, genotypes of the other SNP in the promoter region of SIRT6 had no significant correlation with fat deposition.

**Table 2:** Association of different genotypes of SNPs in the promoter region of SIRT6 with carcass quality traits in Qinchuan cattle

Locus	Genotypes (n)	Intramuscular fat content (%)	Ultrasound loin muscle area (cm)	Backfat thickness (cm^2^)
c.-84 C > T	CC (306)	7.197 ± 0.046	66.216 ± 1.776	1.024 ± 0.018
	CT (196)	7.126 ± 0.058	64.801 ± 1.219	0.943 ± 0.023
	TT (33)	7.212 ± 0.141	62.069 ± 2.209	0.942 ± 0.056
*P*-value		0.507	0.167	0.079
c.-1100 A > G	AA (250)	7.081 ± 0.054^b^	67.473 ± 1.962	0.956 ± 0.020^b^
	AG (217)	7.148 ± 0.058^ab^	63.922 ± 2.106	0.970 ± 0.022^b^
	GG (68)	7.494 ± 0.103^a^	62.824 ± 2.763	1.171 ± 0.038^a^
*P*-value		0.035	0.062	0.018

^a,b^ Means with different superscripts are significantly different (*P* < 0.05)

### Haplotype association analysis

The estimated values of D’ and *r*^2^ were used for the assessment of the relationship between the c.-84 C > T and c.-1100 A > G. The values of D’ and *r*^*2*^ were 0.147 and 0.021, respectively. Previous studies stated that, LD was strong enough when the *r*^2^ values were beyond 0.33 [15]. Therefore, the findings suggested that LD was weak between the two SNPs. It could be argued that recombination will exhibit the opposite trend in genovariation-dense regions.

Four haplotypes were detected and termed as Hap1 to 4 (Table 3). Among the estimated haplotype, frequencies of Hap1 (-CA-), Hap3 (−TG-), Hap4 (-CG-) and Hap2 (−TA-) were 64.50%, 22.00%, 11.00% and 2.50%, respectively. In this study, combinations with frequencies less than 5.0% were excluded for the lack of statistical significanc. As displayed in Table 4, there were higher intramuscular fat content and backfat thickness for individuals with the diplotypes Hap1/4 and Hap4/4 than those with diplotypes Hap1/1 and 1/3 (*P* < 0.05) in Qinchuan cattle. The results suggested that the diplotype Hap1/4 and Hap4/4 in fat deposition were prior to other combinations.

**Table 3:** Haplotypes and their frequencies in the SIRT6 gene for the SNPs

Haplotype	c.-84 C > T	c.-1100 A > G	Frequency
Hap1	C	A	0.645
Hap2	T	A	0.025
Hap3	T	G	0.220
Hap4	C	G	0.110

**Table 4:** Associations between the SIRT6 diplotypes and fat deposition in Qinchuan cattle

Diplotype (n)	Intramuscular fat content (%)	Ultrasound loin muscle area (cm)	Backfat thickness (cm^2^)
Hap1/1 (235)	7.122 ± 0.051^b^	67.732 ± 2.113^a^	0.967 ± 0.020^b^
Hap1/3 (183)	7.119 ± 0.058^b^	64.550 ± 2.394^ab^	0.937 ± 0.023^b^
Hap1/4 (31)	7.516 ± 0.140^a^	59.347 ± 3.816^b^	1.186 ± 0.055^a^
Hap4/4 (40)	7.487 ± 0.123^a^	62.636 ± 3.125^ab^	1.233 ± 0.049^a^
*P* value	0.012	0.029	0.036

Values with different superscripts within the same column differ significantly at *P* < 0.05 (a, b, c) and *P* < 0.01 (A, B, C)

### Promoter assay

In this study, four haplotypes were cloned, and then luciferase reporter (named pGL3-Hap1 to 4) were constructed to analyze the effect of various haplotypes on the activity of transcription. Those plasmids were transfected in 3 T3-L1 cells and to detect the transcriptional activities. Figure 2 showed that Hap3 haplotype had higher activity than Hap1 haplotype (*P* < 0.05).

## Discussion

The intramuscular fat, one of the four mammalian adipose tissue [16], was located in the epimysium, perimysium and endomysium, and was well correlated with sensory characteristics of beef [17, 18]. Thus, it is necessary to clarify the mechanisms of fat deposition for the improvement of intramuscular fat [19]. Emerging evidence suggested that the SIRT6 gene mainly located at nucleus functioned as deacetylation [9], mono-ADP ribosylation [20], depalmitoylation [21], and demyristoylation [22], thereby affecting both of energy and lipid metabolism in mammal. Hence, we inferred that the carcass quality traits could be mediated by SIRT6 gene in indigenous Chinese cattle .

The results of qPCR revealed that bovine SIRT6 gene mRNA was ubiquitously expressed, in agreement with the previous observations such as mice [19] and humans [23]. Especially, the expression level of SIRT6 gene in various tissues and organs verified that the high expression level of SIRT6 gene existed in liver, kidney, muscle and subcutaneous fat, but the slight expression level existed in lung. Except for muscle, the expression distribution of bovine SIRT6 gene reported here was similar to that seen in with mice [20]. Usually, gene expression levels might, at least in part, parallel well with its corresponding function in animals [24]. Therefore, the bovine SIRT6 was highly expressed in subcutaneous fat tissue. This result implied that this gene might be involved in lipornetabolism.

Nowadays, growing observations indicate that genetic variations in the promoter region can influence economical traits in livestock [25]. Both g.-85 G > T and g.-63 G > A in promoter region of the bovine SIX1 gene were significantly related to body measurements in Qinchuan cattle via modification of several binding sites for transcription factors [26]. The c.-1316 A > G mutation in KDR gene promoter region can increase the activity of transcription, consequently enhance intramuscular fat content in Erhualian pigs [27]. In the present study, two novel SNPs (c.-84 C > T and c.-1100 A > G) were detected in the SIRT6 gene promoter region. Correlation analysis showed significant correlation existed between c.-1100 A > G and fat deposition. The fat deposition of individuals that inherited genotype GG was significantly higher than that of individuals with genotype AA.

Previous studies indicated that mutations tended to occur in promoter regions [28], and impact the transcriptional activity [10]. Thus, we predicted the transcription binding factors corresponding to each SNP using the Genomatix software (*ci* value > 85). No differences existed in transcription factors at the c.-84 C > T locus, consistent with the results showing that genotypes of c.-84 C > T did not affect fat deposition in Qinchuan beef. The prediction suggested that, c. -1100 A and c. -1100 G could in sequence bind in three and four *cis*-acting elements (Table 5). These results suggested that the transcriptional activity of SIRT6 gene might be altered by the SNPs in the promoter region.

**Table 5:** The SNPs in the bovine SIRT6 that alter or are adjacent to the *cis*-acting elements

Locus	Genotype	Transcription factors	Cis-acting elements (Recognition sequence^a^)	Target strand
c.-84 C > T	C	Nuclear respiratory factor 1	cacggCGCAtgcgcctt	(+)
	C	C2H2 zinc finger transcription factors 15	cacggcgcaTGCGcctt	(+)
	C	Mouse Krueppel like factor	cacggcgcatgcgCCTTgcga	(+)
	T	Nuclear respiratory factor 1	cacggCGCAtgcgcctt	(+)
	T	C2H2 zinc finger transcription factors 15	cacggcgcaTGCGcctt	(+)
	T	Mouse Krueppel like factor	cacggcgcatgcgCCTTgcga	(+)
c.-1100 A > G	A	NKX homeodomain factors	agggtTGAGtgctgggagc	(−)
	A	Brachyury gene, mesoderm developmental factor	gagctgatcaGGTGtcagggttgagtgct	(−)
	A	Calsenilin, presenilin binding protein, EF hand transcription factor	gtGTCAgggtt	(−)
	G	Selenocysteine tRNA activating factor	tgcacccgcagcTCCCagcactcaaccc	(+)
	G	NKX homeodomain factors	agggtTGAGtgctgggagc	(−)
	G	Brachyury gene, mesoderm developmental factor	gagctgatcaGGTGtcagggttgagtgct	(−)
	G	Calsenilin, presenilin binding protein, EF hand transcription factor	gtGTCAgggtt	(−)

^a^SNP loci in tables, capital letters are core sequence of the transcription factors, and the letters with *ci* value > 85 are underlined

Haplotypes comprised of unique SNP combinations had the potential to account for more variation than single marker selection for economically important traits [29]. Here, our results showed that the intramuscular fat content and backfat thickness of diplotypes Hap1/4 and Hap4/4 increased highly, compared with Hap1/1 and Hap1/3. Similar observations were found between Hap1/1 and Hap1/4 for ultrasound loin muscle area. Use of the TFSEARCH online database, the current study observed that these specific haplotypes had significant effect on two transcription factor combinations, and a separate assay (dual-luciferase reporter assay) reported transcriptional effects associated with these specific haplotypes. In particular, the activity of haplotype Hap1 was significantly lower compared with the haplotype Hap3; and the fat deposition of the diplotype Hap1/1 and Hap1/4 were larger than those of the diplotype Hap1/3.

Our study showed that the c.-1100 A > G within the SIRT6 gene promoter region could significantly influence carcass quality traits. Results gleaned from this study would be possibly contributed to better breeding plan and policies.

## Conclusions

The current study suggested SIRT6 gene was predomiately expressed in subcutaneous fat, and composed of two SNPs in the promoter region in Chinese indigenous cattle. In addition, the SNP c.-1100 A > G and diplotype Hap4/4 (-CA-CG-) might influence fat deposition as a result of alteration in SIRT6 transcriptional activity. This may contribute to deep insights into genes associated with the adaptation and specialization of beef cattle breeds in China.

## Abbreviations

Hif1α: Hypoxia inducible factor 1, alpha
KDR: Kinase insert domain receptor
LD: Linkage disequilibrium
PPARγ: Peroxisome proliferator activated receptor gamma
qPCR: Quantitative real-time polymerase chain reactions
SIX1: SIX homeobox 1
SNPs: Single nucleotide polymorphisms
SREBP: Sterol regulatory element binding protein
